# Understanding the prevalence and manifestation of anxiety and other socio-emotional and behavioural difficulties in children with Developmental Language Disorder

**DOI:** 10.1186/s11689-023-09486-w

**Published:** 2023-06-15

**Authors:** Annabel Burnley, Michelle St Clair, Rachael Bedford, Yvonne Wren, Charlotte Dack

**Affiliations:** 1 Department of Psychology, University of Bath, Bath, Somerset County UK; 2 Bristol Dental School, University of Bristol, Bristol, Bristol County UK

**Keywords:** Developmental Language Disorder, Specific Language Impairment, Parents, Children, Anxiety, Emotion regulation, Socio-emotional behaviour

## Abstract

**Background:**

It is well-documented that children with Developmental Language Disorder (DLD) have a higher likelihood of experiencing anxiety, as well as other socio-emotional and behavioural (SEB) difficulties. Despite this, there is little consensus as to how these difficulties manifest. This study aims to understand the prevalence of broader SEB difficulties and anxiety, informing intervention development by understanding the relationships between them.

**Methods:**

A mixed-methods, case–control study was conducted. First, an online survey was completed by 107 parents of either children with DLD (“DLD sample”; *n* = 57) or typically developing children (“typical sample”; *n* = 50), aged 6–12 years old. Binary SEB statements informed by previous qualitative work (e.g. “my child requires routine/sameness”; “my child has frequent tantrums”) provided an insight into the prevalence of SEB difficulties in both DLD and typical samples. Validated measures of anxiety, emotion regulation, intolerance of uncertainty, insistence on sameness, family stress and coping mechanisms were also collected. Correlation and mediation analyses were run using these validated measures to understand the manifestation of anxiety in children with DLD in more detail. Qualitative interviews were then carried out with a select panel of survey respondents (*n* = 4).

**Results:**

The DLD sample scored significantly higher on all binary SEB statements than the typical sample: experiencing anxiety (80.7%, *p* < .05), requiring routine and sameness (75.4%, *p* < .001) and emotional dysregulation (75.4%; *p* < .001) were the most common difficulties reported for children with DLD. Using the validated scales, family stress and coping mechanisms were found to only correlate with the manifestation of anxiety in the typical group, not the DLD group. “Intolerance of uncertainty” and “insistence on sameness” were found to fully mediate the relationship between DLD diagnosis and symptoms of anxiety. Parent’s interviews provided contextual support for the analysis, as well as highlighting sensory sensitivities as a focus for future research.

**Conclusions:**

Parents of children with DLD appear to cope well with their children’s complex SEB needs. Intervention focussing on intolerance of uncertainty may help the management of difficulties with anxiety. Behaviours such as insistence on sameness should be investigated further, as potential indicators for anxiety amongst children with DLD.

## Background

Developmental language disorder (DLD) is characterised by difficulties with expressive and receptive language, impacting individuals’ everyday lives and social interactions [1]. It is estimated to affect 7.6% of children [2]. As with other neurodevelopmental disorders (NDDs), the difficulties of children with DLD are well documented to extend to a wide array of socio-emotional and behavioural (SEB) difficulties; however, these children are often omitted from relevant intervention (e.g. [3, 4]).

Children with DLD are estimated to experience six times the rate of anxiety and twice the rate of depression than their typically developing peers [5], as well as experiencing lower self-esteem [6] and increased peer difficulties [7]. Nonetheless, SEB difficulties are not inevitable for children with DLD (social adaptation model; [8]), their language profiles do not explain all SEB difficulties (e.g. [9]) and individuals with DLD experience these difficulties at different times and to varying extents (e.g. [10, 11]). Understanding the manifestation of SEB difficulties in children with DLD is key to developing support programmes to prevent the escalation of these difficulties.

### Potential mediators for anxiety

Attention has turned to understanding the manifestation of SEB difficulties in children with DLD [12–15]; however, few studies have yet to explore the manifestation of anxiety specifically. A recent study suggested that other SEB characteristics, such as emotion dysregulation and low self-esteem, could contribute to the maintenance and exacerbation of symptoms of anxiety, through an ongoing, symbiotic cycle [16]; however, there has been no quantitative assessment of this relationship.

Children with DLD do not have an entirely unique socio-cognitive profile, sharing similarities with children with other NDDs (e.g. interpersonal communication [17]), as well as similarities with neurotypical children (e.g. prosociality; [7]). The evidence base regarding the manifestation of anxiety in both these populations (neurotypical children and children with other NDDs) is larger and more established than that of children with DLD [18]. As such, evidence from such populations without DLD can be used to inform suggestions regarding the manifestation of anxiety in children with DLD. This evidence base indicates a range of factors may influence the manifestation of anxiety: key SEB characteristics include emotion regulation [19, 20], intolerance of uncertainty [21–23] and insistence on sameness [24–26]; other potential factors include family communication and coping styles [27, 28].

#### Emotion regulation

Emotion regulation (ER) refers to the ability to exert control over one’s emotional experience [29] and has an inherent relationship with anxiety; a limited ability to regulate one’s thoughts and emotions can lead to the maladaptive “worry loops” and physical panic characteristic of anxiety [30]. Amongst DLD samples, emotion dysregulation has long been identified as more common than in neurotypical samples (e.g. [31]). Furthermore, aspects of ER (such as emotional awareness) have been shown to protect against developing anxiety in children with DLD [12]; however, the relationship between ER itself (and anxiety) has yet to be studied.

The language profile of children with DLD could be used to explain a potential relationship between ER and anxiety. Regulation strategies typically involve cognitive restructuring to reduce an angry or stressed response or refocussing on stimuli that evoke fewer unpleasant emotions [32]. Both of these strategies require a level of “inner speech” [33], which requires intact language skills and has therefore unsurprisingly been shown to be limited in children with DLD [34]. Furthermore, the difficulties with vocabulary and both use and understanding of grammatical structure in those with DLD can limit their ability to discuss emotions and learn regulation strategies from their caregivers [35]. An inability to regulate your emotional response in stressful situations, or express how you are feeling, may lead to symptoms characteristic of anxiety, such as situational avoidance, panic and somatic problems [36–38].

Research on children with other NDDs (e.g. Autism Spectrum Disorder; ASD) has also identified emotion dysregulation’s role in explaining the manifestation of anxiety (e.g. [19]). Amongst these children, however, this relationship has been explained through their unique socio-cognitive characteristics, rather than simply their communication differences [39]. Examples of these socio-cognitive characteristics include reduced social attention, sensory sensitivities, intolerance of uncertainty, insistence on sameness and situational avoidance, many of which are shared with the DLD population [7, 40]. Given the heterogeneity of the DLD population [41], it is possible that a causational relationship between ER and anxiety could be driven by similar cognitive characteristics outside of their core language differences.

Alternatively, the children’s language profile alone could be the primary contributor to anxiety amongst children with DLD, with less influence from ER. Indeed, social communication deficits have been found to explain much of the development of anxiety amongst neurotypical children [42]. Whilst children with DLD share some of these important socio-cognitive similarities with both neurotypical children and children with other NDDs, they remain distinct populations, rendering an independent investigation into the relationship between ER and anxiety necessary.

#### Intolerance of uncertainty and insistence on sameness

Intolerance of uncertainty (IU) is a trait characterised by preference for predictability and reacting with emotional distress when presented with the unknown or unfamiliar [23]. It is recognised as a transdiagnostic risk factor in the formulation of a number of anxiety disorders [43–45]. Children with DLD could be suggested to have further vulnerability to IU, as their language difficulties render instructions and cues difficult to understand [46]. Furthermore, any expressive difficulties increase the risk of being misunderstood themselves, limiting their control over an interaction [46]. As a result, they are likely to experience increased uncertainty in everyday situations, thus increasing their vulnerability to anxious thinking [47]. Indeed, IU has been identified more consistently as a co-occurring experience amongst children with other NDDs, who share similar difficulties in inferring meaning from interactions as those with DLD (e.g. ASD and Williams syndrome [48]). Moreover, it has been suggested to contribute to the manifestation of their symptoms of anxiety [49–51], prompting the development of tailored interventions for children with other NDDs, which have yet to be designed to support children with DLD (e.g. [52]).

The specific presence of IU has only recently been identified amongst children with DLD [16]. In this sample, children with DLD were found to require additional preparation for unfamiliar situations and experienced emotional distress at unexpected changes in routine [16]. The relationship between anxiety and IU could be suggested to be particularly strong regarding social anxiety disorder (SAD), given both the level of social ambiguity experienced amongst individuals with DLD [40], paired with their keen social attentiveness [48]. Indeed, a directional relationship between IU and SAD has been found in neurotypical samples, but only in cases where IU results in avoidance behaviours, rather than when uncertainty is perceived as “unfair” [53]. Should SAD manifest in children with DLD in much the same way as it does for typically developing children, it is possible that IU could also explain many of the documented social avoidance behaviours recognised amongst individuals with DLD [7].

Insistence on sameness is characterised by a preference for familiarity, routines and a behavioural inflexibility [54] and is conceptually linked to IU. Where IU is a form of cognitive rigidity in which unexpected events are perceived as stressful, insistence on sameness is a behavioural attempt to minimise uncertainty through increasing familiarity [55]. By conception, insistence on sameness is considered a defining feature of ASD [56]; however, it has also been recognised as a characteristic amongst populations with other NDDs [54, 57] and language disorders [58]. Furthermore, it has been suggested to not only result from IU [49] but also contribute to anxiety [24, 25, 59, 60].

A reliance on routine and preference for familiarity has been described amongst children with DLD in a recent qualitative investigation [16], as has children’s reported need to “impose structure” on their social interactions [40]. This was understood by parents and teachers to result from the children’s attempts to increase predictability and personal autonomy when much of their day to day felt out of their control [16]. Understanding more about the relationship between anxiety, IU and insistence on sameness could aid intervention efforts or help identify children with DLD at risk of developing anxiety. First, however, these phenomena need to be identified through quantitative analysis; an investigation that has yet to be conducted.

#### Influence of the family environment

Child temperament has long been linked to the family environment (e.g. [61]): the socio-emotional context of each family unit, as influenced by the behaviour and quality of the interpersonal relationships within the household (Bowen Family Systems Theory; [62]). Raising a child with DLD has been shown to increase family stress [63, 64], which has been linked to an increase in the child’s experience of anxiety [64]. This could be explained through Sameroff’s unified theory of development [65], where it is proposed that much of a child’s ER is directed by the behaviour of those around them. As a result, when those in the family system display SEB difficulties, the child may internalise these and learn less adaptive ways of managing their own emotions [66]. This could only be further exacerbated by the recognised heterogeneity of DLD [41].

More specifically, family communication styles have been shown to be key in adaptive SEB development of children, particularly for those with developmental disorders [67]. A focus on open communication and affirmation in adverse circumstances has been shown to increase SEB resilience [67, 68]. However, increased family stress is suggested to impact the different parenting practices and coping strategies used within the household. For example, stressed parents have been suggested to model more “anxiety-enhancing” coping styles, such as avoidance and control (e.g. [69]), and maladaptive coping styles that are then modelled to the child. Should family factors such as these be conclusively linked with child SEB development, it suggests a focus for a whole-family intervention (e.g. Triple P: [70]).

### The current study

Whilst anxiety and emotion dysregulation have been previously identified in children with DLD, the relationship between them has yet to be fully understood. Given the socio-cognitive similarities that children with DLD share with both neurotypical samples and individuals with other NDDs, research is required to understand whether the manifestation of anxiety is driven by similar factors. Measuring characteristics such as ER, IU, insistence on sameness and family context will help provide a deeper understanding of the manifestation process.

The evidence-base for the psychopathology amongst DLD populations is currently primarily quantitative (e.g. [64, 71–73]), missing the potential for novel insights that could be uncovered by qualitative discussion with the participants themselves. Furthermore, qualitative insights can be used to bridge the gap between research and practice [74, 75], particularly when the perspectives of those with language disorders are less readily acknowledged [18]. Therefore, the current study takes a mixed-methods approach, combining the scientific objectivity of quantitative analysis, with the participant-focussed contextual insights of qualitative analysis [76, 77]. Similar methods have been used with participants with other NDDs, leading to a further understanding of the potential relationship between anxiety, IU and ISS (e.g. [49]).

The first aim of the current study is therefore to test the hypothesis that children with DLD report higher rates of all stated SEB difficulties than those of typically developing children. Secondarily, this study aims to understand the roles ER, IU and insistence on sameness, and family context may play in the manifestation of anxiety. This second aim will be explored through correlational and mediation analyses. Qualitative interviews of parents of children with DLD will provide further context for all quantitative analysis.

## Methods

### Design

This study used a mixed-methods, explanatory sequential design [78]; quantitative data is first collected and analysed, before a second phase of qualitative data collection and analysis [79]. This is due to the relatively unexplored nature of the topic area, as well as the recognised demographic biases in quantitative data sampling (e.g. [80]). This method supports an inclusive research approach, whereby the participants themselves are consulted regarding interpretations (e.g. [49]).

A case–control quantitative survey study was used to explore the prevalence of SEB difficulties and the relationships between them. On finalising this stage of data collection and analysis, any demographic sampling biases were identified (e.g. over-recruitment of white, high-income participants [81]), as well as any additional questions that emerged from the findings. Phenomenological qualitative interviews were then held with a small and separate sample of parents of children with DLD. Purposeful recruitment was used to ensure a range of life contexts was captured (including race, income and relationship status), and the quantitative findings were discussed with them individually. A joint display has been used when reporting results [82]; qualitative findings are integrated throughout the quantitative analysis, to provide additional insight and context.

### Participants

Participants who completed the online survey (“survey sample”) were recruited between January and April 2022; those who completed the interview (“interview sample”) were recruited between June and July 2022. The survey sample was recruited through convenience sampling and the interview sample through purposeful sampling. Convenience sampling was chosen for the survey sample due to the expected difficulty in recruiting, given the low level of awareness of DLD [18]. Purposeful sampling was chosen as a way to recruit parents from populations that were demographically underrepresented in the survey sample, to ensure representation of a range of family contexts and increase the validity of the results [83]. Participants were first recruited through advertising to the engage with DLD database (E-DLD; *engage with DLD * [84]), an online network connecting families with DLD with researchers. Research adverts were also circulated via Twitter, Mumsnet and relevant parenting groups on Facebook. Parents from the interview sample had not completed the survey but were recruited purposively from previous research [16].

The survey sample comprised a total of 107 parents of children with DLD (“DLD sample”; *n* = 57; male children *n* = 29) and typically developing children (“typical sample”; *n* = 50; male children *n* = 27). To be eligible, children had to be aged between 6 and 12 years old (*M* = 8.92; *SD* = 1.76); participants were excluded if they failed to complete the first “parent-informed SEB statements” question. Participants in the DLD sample were also required to confirm their child’s DLD diagnosis through self-report. Due to the expected range of both nationalities and services accessed to receive a diagnosis, no uniform diagnostic report was required. Parents responded to the following questions: “Do you have a child that has difficulties either using spoken language or understanding spoken language?” (answer response “yes” was required for the DLD sample) and “Is there a known explanation for this language difficulty (that is NOT DLD/specific language impairment)?” (answer response “no” was required for the DLD sample).

Child and parent age, as well as child sex and parent’s self-reported gender, was collected. Country of residence, parent’s ethnicity and number of children in household were also confirmed. To provide an indication of socioeconomic class, participants’ postcodes, monthly income and the occupation of main income earners were also asked. Characteristics of the survey sample can be found in Table 1; participants were predominantly white, mothers and had above average monthly income (c.2,650 GBP in March 2022; [85]). The only significant difference between the DLD and typical samples was for parent self-reported gender; nearly all parents who completed the survey for the DLD sample were mothers (92.7%), whereas statistically more fathers completed the survey for typically developing children (36.7%; *p* < 0.001).

**Table 1:** Demographic characteristics of the Developmental Language Disorder sample and typically developing sample who completed the online survey

		Typical sample (*n* = 50)		DLD sample (*n* = 57)	
Child gender, % (*N*)	*Male*	58.7	27	51.8	29
	*Female*	41.3	19	48.2	27
Child age, M (SD)		8.9	1.7	9.0	1.8
Parent gender, % (*N*)	*Male*	36.7*	18	5.5*	3
	*Female*	63.3*	31	92.7*	51
Ethnicity, % (*N*)	*Black*	2.0	1	-	0
	*Hispanic*	-	0	1.8	1
	*South Asian*	6.0	3	1.8	1
	*White*	90.0	45	96.5	55
	*Other*	2.0	1	-	0
Monthly income (GBP), M (SD)		3332.9	1387.5	3096.9	1314.9
Number of children, M (SD)		2.1	0.7	2.2	1.0

^*^Significant difference between groups at *p* < .008 (Bonferroni adjustment)

The interview sample comprised of four parents of children with DLD, each of whom had completed the survey prior to interview; two mothers were white British, one white European (first-generation immigrant to the UK; bilingual family) and one Black British. Their incomes ranged from low income (£20,000 per annum) to high income (above £60,000 per annum) and lived across England, Scotland and the Isle of Wight. Three of the parents were cohabiting, and one was a single-parent household, without support from the father. Their children were 6, 7, 10 and 11 (see Table 2). All information gathered was self-report, including diagnosis.

**Table 2:** Characteristics of the mothers interviewed and their children with Developmental Language Disorder

Parent pseudonym	Child pseudonym	Age of child	Child gender	Income^a^	Ethnicity	Number of siblings	Known comorbidities	Relationship
Josephine	Femi	6	Male	Low	Black British	0	-	Single
Liz	Ali	7	Female	ND	White British	2	-	Cohabiting
Iris	Dimo	10	Male	ND	White European	1	-	Cohabiting
Helen	Joe	11	Male	High	White British	1	Dyslexia, APD, PDA traits^b^	Cohabiting

*PDA* pathological demand avoidance, *APD* auditory processing disorder, *ND* not disclosed

^a^Income groups are defined as follows: “low”, below £30,000 per annum; “middle”, £30,000–£60,000 per annum; “high”, above £60,000 per annum. ^b^No autism spectrum disorders

### Measures

#### Parent-informed SEB statements

**Fig. 1:**
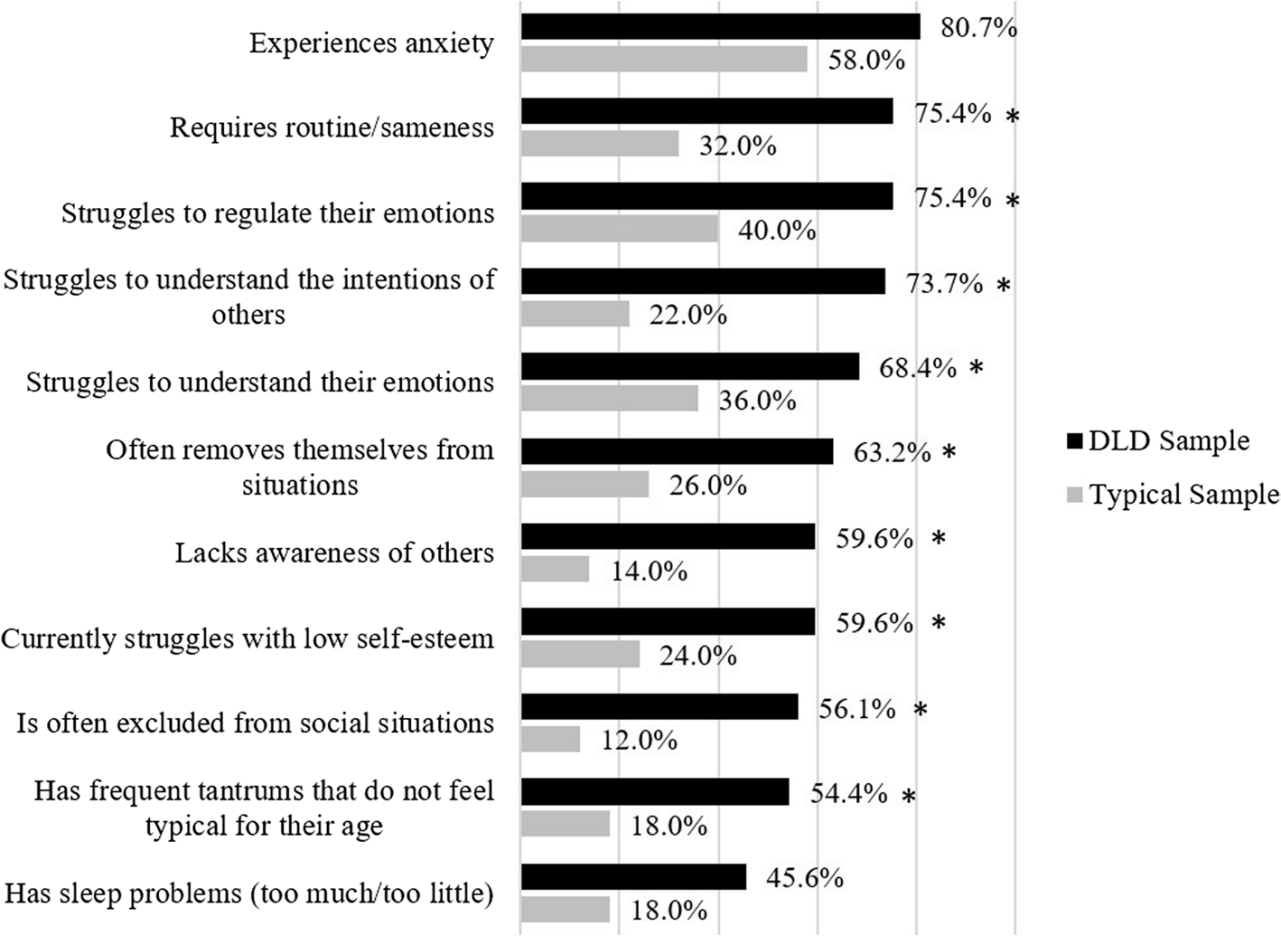
Difference between groups on parent’s self-report of their child’s psychosocial difficulties *Significant difference between groups at *p* < .005 (Bonferroni adjustment)

Eleven statements were derived from previous work according to the SEB difficulties described to be bothering parents of children with DLD the most [16]. These were presented to parents at the beginning of the survey; they were asked “Is it true that your child…” and were required to choose between “true” or “false” to each statement. The full list of statements can be found in Fig. 1 (e.g. “…struggles to understand their emotions”). For all statements, a higher score indicates experiencing more difficulties.

#### Anxiety

Anxiety was measured using the parent-report version of the Spence Children’s Anxiety Scale (SCAS-P; [86]. To minimise the length of the survey, only the statements relating to subscales of interest were used (also regrouped in prior literature; e.g. [87]). These were determined from anxiety disorder symptoms found to be common in children with DLD (e.g. [16]): generalised (GAD), social (SAD) and separation anxiety, as well as obsessive compulsive disorder (OCD; e.g. “my child worries that something bad will happen to them”, “my child worries what other people will think of them”, “my child worries about being away from us/me”, “my child has to keep checking that they have done things right”, respectively). Questions use a 5-point Likert scale, with answers ranging from “never” to “always”. Higher scores indicate an increased experience of anxiety. The scale has well-established reliability in both neurotypical children (e.g. [88]) and those with NDDs (e.g. [89]). Using Cronbach’s α, internal reliability was very strong for both case–control samples (DLD group α = 0.94; typical group α = 0.92) and acceptable across all subscales (DLD group α = 0.72–0.89; typical group α = 0.75–0.84).

#### Intolerance of uncertainty

The short form of the intolerance of uncertainty scale was used (IUS-12; [23]). It is a refined version of the 27-item scale and requires parents to indicate how characteristic 12 separate statements are of their child (e.g. “unforeseen events upset my child greatly”, and “a small unforeseen event can spoil everything, even with the best of planning”). Response options are provided along a 5-point Likert scale, from “Not at all characteristic of my child” to “Entirely characteristic of my child”. Higher scores indicate higher levels of IU. In neurotypical samples, the scale has been found to have a good test–retest reliability, as well as construct validity against other symptom measures of anxiety (e.g. [90]). Internal reliability was very high for both case–control samples (DLD group α = 0.99; typical group α = 1.00).

#### Insistence on sameness

The 15-item “insistence on sameness” subscale (ISS) of the Repetitive Behaviour Questionnaire (RBQ) was used [91]. Items are rated on either a 4-point scale or a 3-point scale, depending on the behaviour, for example “Are there any aspects of routine that your child insists must remain the same?” would be answered “no”, “mild problem” or “serious problem”, and “How do they react if any changes are made to their surroundings at home?” would be answered “may comment on”, “accepts the change”, “will accept the change, but shows extreme anxiety”, or “will not accept the change”. There are two subdomains: “inhibitory” which refers to avoidance of uncertainty and paralysis in the face of it and “prospective” which refers to desire for predictability and the active seeking of information to increase certainty of future events. Higher scores indicate a higher level of intolerance of uncertainty. Although most widely used in populations with ASD, showing good construct validity (e.g. [92]), the scale has also been used in both neurotypical samples (e.g. [93]), as well as samples with language difficulties (that include DLD; e.g. [94]). Internal reliability was high for both case–control samples in this study (DLD group α = 1.00; typical group α = 1.00).

#### Emotion regulation

A total score was derived from five items taken from the Child Social Behavioural Questionnaire (see [95]: “shows mood swings”, “gets over excited”, “gets easily frustrated”, “gets over being upset quickly” and “acts impulsively”. Each of these items were rated on a scale from “almost never” to “almost always”. Higher scores indicate a higher level of emotion dysregulation. Reliability and construct validity have been demonstrated in previous psychometric work [96]. Due to the small number of items in the scale, inter-item correlations were used to measure reliability [97]. Correlations were high between all items (range = 0.90–1.00, *M* = 0.96).

#### Parenting stress

The Parenting Stress Scale (PSS; [98]) was used, for which parents are required to indicate how much they agree with 18 statements along a 5-point Likert scale (from “strongly disagree” to “strongly agree”). Examples of these statements include “I sometimes worry whether I am doing enough for my children” and “It is difficult to balance different responsibilities because of my children”. Higher scores indicate a higher level of experienced parenting stress. The scale is widely used and has been validated in populations from Hong Kong [99] to Spain [100]. Internal reliability was excellent for both case–control samples in this study (DLD group α = 1.00; typical group α = 1.00).

#### Family communication style

The Family Problem-Solving Communication Index (FPSC; [101]) was used as a way of measuring communication styles. It is a 10-item instrument, for which the parent is required to indicate to what extent the item reflects their family’s pattern of communication on a 4-point Likert scale ranging from “false” to “true”. There are two subscales: “affirming communication”, for example “we take the time to hear what each other has to say or feel”, and “incendiary communication”, for example “we walk away from conflicts without much satisfaction”. These subscales have good convergent validity [101], and the internal reliability was high for both “affirming” (DLD group α = 1.00; typical group α = 1.00) and “incendiary” subscales (DLD group α = 1.00; typical group α = 1.00).

#### Family coping mechanisms

The Brief-Coping Orientation to Problems Experienced (Brief-COPE) is a shortened version of the original 60-item scale [102]. Participants are required to respond to 28 items on a 4-point Likert scale, from “I haven’t been doing this at all” to “I’ve been doing this a lot”. The measure has been widely validated in both clinical populations and cross-culturally (e.g. [103, 104]). Items are traditionally grouped into three categories of coping styles: “Problem-focussed”, “Emotion-focussed” and “Avoidant coping” [105]. However, for the sample in this study, internal validity was low for certain subscales (emotion-focussed, DLD group α = 0.001; problem-focussed, DLD group α = 0.14, typical group α = 0.07). As such, a factor analysis was run to redefine three new subscales: “adaptive coping” was characterised by items concerning acceptance, positive reframing and problem-oriented action (e.g. “I’ve been taking action to try to make the situation better”); “maladaptive coping” was characterised by avoidance and negative reframing (e.g. “I’ve been criticizing myself”); and “alternative coping” was characterised by drug taking and spirituality (e.g. “I’ve been praying”, or “I’ve been using alcohol to make myself feel better”). Higher scores for each subscale indicate use of that coping mechanism; each coping style is not mutually exclusive. Statements that were excluded from these groups due to low correlation values included the following: “I’ve been expressing my negative emotions”, “I’ve been getting help and advice from other people” and “I’ve been making fun of the situation”. As a result, the validity of the final subscales was high for both DLD (*α* = 0.68–0.81) and typical groups (*α* = 0.65–0.83).

### Procedure

Ethical approval for the study was granted by the Psychology Research Ethics Committee at the University of Bath (Ref: 21–215). An online survey was used as the method for data collection. Participants were invited to take part through an online advert, which included a WebLink to the consent form and survey. In the advert, they were also given the option to contact the research team by email to ask any questions. The survey took 15–20 min to complete, including online consent and debriefing. Statements within each question were randomised, whilst the questions themselves were in a fixed order, appearing in the same order in which they are listed in the “Measures” section. This was important to minimise bias in reporting order (e.g. due to satisficing; [106]). Parents were entered into a prize draw to win a £50 voucher to compensate them for their time.

**Fig. 2:**
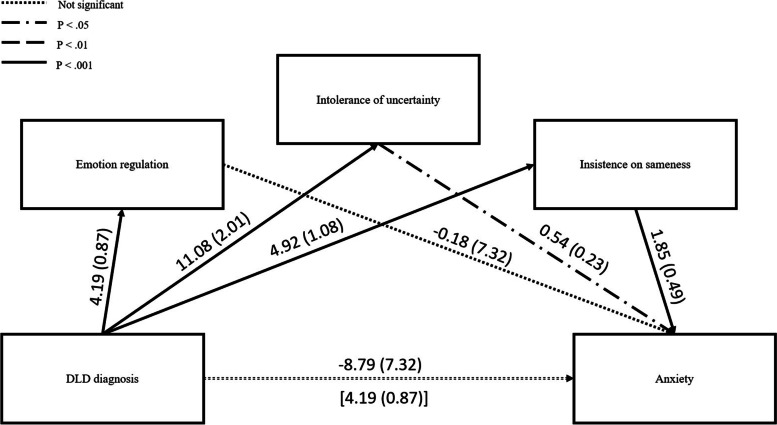
Visual representation of the direct and indirect effects of emotion regulation, intolerance of uncertainty and insistence on sameness in mediating the relationship between DLD diagnosis and anxiety

Interviews were conducted online using Microsoft Teams, lasting up to 40 min each. Conversation began with an overview from the parents of their child’s key SEB difficulties, to provide the researcher with context for further discussion. Then parents were presented with the results from the survey (Figs. 1 and 2). Discussion topics included the following: (1) whether they personally identified with the results, (2) whether they were surprised by any of the findings and (3) provided them with an opportunity to share lived experiences that either expanded on the results or conflicted with them.

### Data analysis

Analysis was completed using IBM SPSS Statistics for Windows Version 26.0. Full data (i.e. those that completed all measures; “complete sample”; *n* = 79) and incomplete data (i.e. those that omitted at least one measure; “incomplete sample”; *n* = 28) were compared to identify any potential biases created by missing data; categorical variables were compared using chi-squared tests (parent gender, child sex, ethnicity); and continuous variables were compared using independent *t*-tests (age, income, anxiety symptom scores). Amongst the incomplete sample, those who completed the child anxiety symptom measure (*n* = 21) demonstrated significantly lower scores (*M* = 10.38, *SD* = 13.0) than those with full data (*n* = 79; *M* = 20.1, *SD* = 14.1; t (98) = 2.85, *p* = 0.003). This difference was most significant amongst the typical sample (full sample: *M* = 13.6, *SD* = 9.6; incomplete sample: *M* = 1.9, *SD* = 4.5; t (46) = 3.36, *p* < 0.001). There were no other significant differences on any demographic measures: income (t (78) = 0.43, *p* = 0.67), child sex (*χ*^2^ (1) = 0.28, *p* = 0.60), child age (t (98) = 0.86, *p* = 0.20), parent gender (*χ*^2^ (2) = 1.06, *p* = 0.59) and ethnicity (*χ*^2^ (4) = 7.42, *p* = 0.12). DLD and typical samples were also compared; the same tests were used (independent samples *t*-tests and chi-squared tests) across the same demographic variables (child age, income, anxiety symptom scores, parent gender, child sex, ethnicity). A significantly higher proportion of fathers was found in the typical sample than the DLD sample (*χ*^2^ (2) = 16.30, *p* < 0.001); no significant differences were found between samples for any other demographic characteristics (*ps* = 0.80–0.34; see Table 1).

To estimate the prevalence of SEB difficulties in both children with DLD and typically developing children, the “parent-informed SEB statements” were compared between groups (typical vs. DLD), using chi-squared tests. Then, to quantify any differences in individual scores (e.g. anxiety, emotion regulation), the rest of the validated SEB symptom and coping scales were compared between groups using independent samples *t*-tests, for all subscales as well as total scores. All tests were run with Bonferroni-adjusted significance levels.

The potential relationships between SEB characteristics were explored through Pearson correlations which were run between all demographic variables and validated SEB symptom and coping scales (with Bonferroni-adjusted significance levels). To then determine the role of key SEB characteristics in contributing to the manifestation of anxiety (SCAS-P), a parallel mediation model was run using the PROCESS macro in SPSS [97]. The dichotomous variable of sample group (i.e. typical or DLD sample) was used as the independent variable; the total anxiety score (SCAS-P) was used as a continuous dependent variable in the model. Variables that correlated significantly with anxiety for the DLD sample (i.e. IUS, ISS, ER, family coping or parent stress measures) were then included as potential mediators. Bias-corrected and accelerated 95% confidence intervals were used, alongside 10,000 bootstrap resamples to adjust for measurement error. The Sobel test was used to identify the significance level of any mediation effect.

To explore any identified relationships further, the qualitative interviews were conducted and then analysed using interpretative phenomenological analysis [107]. This approach was chosen due to the participant-centred nature of the conversations and the focus on the phenomenon of raising a child with DLD [107]. Stories and quotes were used to either expand on or contrast with the quantitative data; pseudonyms were used throughout. Subsequently, data from the qualitative and quantitative phases were integrated. The result is a joint display under the key study aims, demonstrating how each methodology compliments one another [108].

## Results

### Aim 1: Socio-emotional and behavioural difficulties

The DLD sample scored significantly higher on all parent-informed SEB statements than the typical sample (*ps* < 0.05; see Fig. 1). The majority of both the DLD sample and typical sample reported their children “experiencing anxiety” (*DLD* = 80.7%; typical = 58.0%). Only a minority of the typical sample reported their child experiencing any of the other SEB difficulties listed (12.0–40.0%). In comparison, a majority of the DLD sample reported their child experiencing all but one of the SEB difficulties (54.4–80.7%): “sleep problems” (45.6%).

The largest differences between DLD and typically developing sample are “struggles to understand the intention of others” (difference = 51.7%; *χ*^2^ (1) = 28.46, *p* < 0.001), “lacks awareness of others” (difference = 45.6%; *χ*^2^ (1) = 23.48, *p* < 0.001), “is often excluded from social situations” (difference = 44.1%; *χ*^2^ (1) = 22.66, *p* < 0.001) and “requires routine/sameness” (difference = 43.4%; *χ*^2^ (1) = 20.32, *p* < 0.001).

#### Qualitative insights

All mothers agreed that anxiety, routined behaviours and emotion dysregulation were the most problematic for their child. Parents particularly described anxiety behaviourally, through incessant worrying, nail biting (Ali, 7 years old) and seeking reassurance and company:

Even now if I’m making dinner in the kitchen [Femi] just has to come and stand beside me, as he feels like he hasn’t been around me that much, if he’s been playing in the living room or whatever… and then if I’m cleaning the kitchen afterwards, he’s just like ‘when are you going to come and sit with me, when are you coming’. So yeah he likes someone to be present with him and I would say that’s a bit of a part of anxiety for him – he doesn’t like being by himself. (Josephine; Femi, 6 years old)

Iris described how Dimo (10 years old) has learnt to manage his worries through the counselling he has accessed over the last year:

He has learnt he can overcome situations, there is no need to be anxious all the time. [The worries] certainly have not gone. I think he always will be a worrier, but he has absolutely learnt to deal with them better… he tends to brood and to dwell on things, so he needs to be snapped out of it, either by himself, or if you tell him. So, he is really making that effort not to brood… since he has learnt that it’s okay to talk about it, he now increasingly comes to me when there is a worry. So he is not just bottling it up.

Preference for routine was experienced by all children of the mothers that were interviewed; however, the reasons behind this appeared to differ. Josephine suggested “requires routine and sameness” would come equal to “anxiety” for her 6 years old, as she feels they are so inter-linked: “A lot of [Femi’s] routined behaviours can be a ‘worry’ thing”. Similarly, Liz noticed that when anything at school changes, or there is a change to Ali’s routine (7 years old), there is huge upset, and she focusses on tidying her room and ordering her teddies to make her feel better:

Anything out of the norm that she’s not fully sure of what’s happening, or what it will look like… She’s started to worry about finishing this year and going back up to a new teacher in the year above… I know now if she’s organising her toys I just call school and say we’re going to be late. You can’t rush her or stop her, otherwise there’s a full-blown meltdown.

For Iris, she felt Dimo (10 years old) simply found routine easier. He did not have any emotional response to routine changing but was simply more likely to forget what he was meant to be doing: “He’s happy to go along with whatever, but the thing he struggles with is to remember a change in routine… it just goes over his head”.

Both Helen and Iris were surprised to see “lacks awareness of others” on the list. They both described their children as “actually very empathetic”, describing how:

There was a new child that joined [school] during or after Covid, so a very tricky situation for that child, and it has been specifically pointed out that it was Dimo that was specifically going over to that child and making sure they were included and welcomed. I was over the moon. (10 years old).

Helen thought perhaps this lack of awareness of others was in fact lack of self-awareness:

[Joe’s] very aware of other people if they are sad, but he’s totally unaware of the impact he has on other people… for example, if I had a row with his sister, he would be completely able to observe that and understand what had happened and why we might be upset, but he basically has zero self-awareness… in a social situation with peers he makes odd noises and sort of talks to himself… he literally couldn’t care less that other people might think that’s a bit odd (11 years old).

Similarly, Iris and Josephine questioned whether or not the “struggle to understand the intention of others” could be due to the relative immaturity of their children, when compared to their typically developing peers. Josephine described how it is others’ deceit that Femi (6 years old) particularly struggles to understand:

At school he’s got in trouble before from doing something that he knows he’s not supposed to do it, but because other people are, and they’re his friends, he’s just like ‘yeah but my friends are doing it’, and the teacher says, ‘but is that right’, and he says ‘no, but my friends are doing it’… there’s no connection there at all…I know his friends know that that’s wrong and they’re trying to get him to follow, and he will just follow. He’s quite a sheep.

Helen was surprised to see that “is often excluded from social situations” was so low down. She described how last summer she “invited every child possible for a playdate, and it just never gets reciprocated”. She also described how this has only become more challenging as Joe (11 years old) has become older; it is no longer up to the parents to organise playdates, and the children invite who they want, meaning Joe is always left out.

The two mothers of the younger children (Josephine and Liz; 6 and 7 years old, respectively) mentioned being surprised that “sensory sensitivity” was not on the list of SEB difficulties. Josephine described how Femi “Always has to be touching things, I’ve had to get him a lot of fidget things for him to play with, to keep him occupied… it’s the physical touch, he’s always wanting to feel people”. He also appeared to prefer noise and loudness, for example loud crowds rather than quiet streets. Lack of auditory stimulation made Femi feel “uncertain”:

He likes constant noise, and something going on… he stomps around constantly… he always has to have some sort of noise, the volume control in his voice sometimes, I’m right beside him, I don’t know why, I find it so funny, he’s just like ‘AND THEN!’, I say ‘mummy can hear you, can we maybe bring it down a little bit… but then it goes down like 0.5%” You have to keep asking him. (6 years old).

In contrast, Ali (7 years old) has become very selective with her clothing and will now only wear soft tracksuit bottoms and tops, no socks and does not like the feeling of her school shirt tucked into her skirt. These parents wondered whether it might be a “comfort thing” (Josephine; Femi, 6 years old), in response to feeling anxious or uneasy, though it was hard to pinpoint.

### Aim 2: Understanding the manifestation of anxiety

The DLD group had significantly higher rates of anxiety on all subscales compared to the typical sample. The largest significant difference was for social anxiety (*p* < 0.003, see Table 3) and separation anxiety (*p* < 0.003), followed by generalised anxiety (*p* < 0.003). Regarding the potential mediators for anxiety, the DLD group had significantly higher rates of IUS on both subscales than the typically developing group (*ps* < 0.003) as well as emotion dysregulation (*p* < 0.003) and insistence on sameness (*p* < 0.003), all with large effect sizes (*d* = 1.00–1.18). There was no significant difference across groups for parenting stress (*p* = 0.40), communication styles (*ps* = 0.17–0.18) or either maladaptive (*p* = 0.13) or alternative coping (*p* = 0.27). Parents of the DLD group did, however, report significantly higher use of adaptive coping styles than those of typically developing children (*p* < 0.003).

**Table 3:** Differences between groups on symptoms of anxiety, intolerance of uncertainty, emotion regulation, insistence on sameness, parent stress and family-coping styles

		Typical sample		DLD sample		Effect size
		*M (SD)*	*Range*	*M (SD)*	*Range*	*Cohen’s d*
SCAS-P	*Total*	11.7 (10.0)	0–50	23.9 (15.3)	0–54	0.94*
	*GAD*	3.2 (2.8)	0–12	6.1 (4.5)	0–16	0.76*
	*SAD*	3.6 (3.3)	0–13	8.0 (5.3)	0–18	0.98*
	*OCD*	1.5 (2.9)	0–14	2.8 (3.0)	0–13	0.43
	*Separation*	3.3 (3.1)	0–12	7.1 (5.2)	0–18	0.88*
IUS	*Total*	19.6 (6.7)	12–39	30.3 (10.8)	12–55	1.18*
	*Prospective*	11.8 (4.3)	7–24	17.9 (6.9)	7–32	1.05*
	*Inhibitory*	7.8 (3.1)	5–17	12.6 (4.8)	5–25	1.17*
EmReg	*Total*	10.9 (3.3)	7–21	15.1 (4.7)	7–24	1.05*
ISS	*Total*	2.3 (3.1)	0–18	7.2 (6.2)	0–24	1.00*
PSS	*Total*	37.3 (9.5)	21–63	37.9 (11.3)	18–61	0.06
FPSC	*Affirming*	11.7 (2.3)	5–15	12.2 (2.5)	5–15	0.21
	*Incendiary*	4.2 (2.7)	0–9	4.8 (2.9)	0–13	0.20
BCOPE	*Adaptive*	21.0 (4.7)	9–30	25.4 (5.6)	13–34	0.85*
	*Maladaptive*	13.2 (4.0)	8–28	14.2 (3.8)	8–29	0.24
	*Alternative*	6.7 (1.7)	5–11	6.4 (2.5)	2–16	-0.14

NB, the comparator group is the typical sample, meaning a positive *d*-value indicates the Developmental Language Disorder (DLD) group displayed more of those symptoms

^*^Significant difference between groups at *p* < .003 (Bonferroni adjustment)

#### Correlations

Table 4 presents the Pearson’s correlations between age, income, validated SEB symptom scales (including anxiety) and family coping scales for the DLD and typical samples.

**Table 4:** Pearson correlations between study variables for children with DLD (bold), and typically developing children (italics)

	Age	Income	GAD	SAD	OCD	Separation	Anxiety total	IUS total	Emotion regulation	RBQ-ISS	PSS	FPSC affirming	FPSC incendiary	BCOPE adaptive	BCOPE maladaptive	BCOPE passive
**Age**	--	-*.18*	*-.11*	*-.02*	*-.02*	*-.25*	*-.12*	*.00*	*-.03*	*-.04*	*-.20*	*.37*	*-.19*	*-.17*	*-.34*	*-.18*
**Income**	**.08**	--	*-.07*	*-.20*	*-.10*	*.11*	*-.09*	*-.34*	*-.35*	*.17*	*.07*	*-.07*	*.03*	*-.05*	*.00*	*.18*
**GAD**	.36*	-**.10**	--	.68*	.77*	.63*	.92*	.60*	*.14*	.65*	*.21*	-.41*	*.23*	*.33*	.49*	*-.01*
**SAD**	.39*	-**.13**	.73*	–	.47*	.61*	.84*	.68*	*.36*	.57*	*.29*	-.40*	*.17*	*- .01*	.53*	*-.05*
**OCD**	**.23**	**.09**	.54*	.48*	--	.39*	.78*	.50*	*.11*	.77*	*.27*	*-.28*	*.17*	*.24*	.48*	*-.02*
**Separation**	**.15**	-**.13**	.69*	.70*	.47*	--	.79*	.48*	*.12*	.54*	*.11*	-.49*	*.25*	*.31*	.44*	*.07*
**Anxiety total**	**.34**	-**.10**	.89*	.89*	.68*	.88*	--	.70*	*.23*	.79*	*.27*	-.49*	*.25*	*.27*	.06*	*.00*
**IUS total**	**.25**	**-.09**	.56*	.70*	.59*	.63*	.74*	--	*.34*	.55*	*.11*	-.43*	*.37*	*.33*	.53*	*-.03*
**ER**	**- .08**	-**.21**	.48*	.46*	**.33**	.63*	.59*	.63*	--	*.27*	*.38*	*-.33*	.44*	*.11*	.42*	*-.04*
**RBQ-ISS**	**.15**	-**.14**	.49*	.55*	.61*	.56*	.65*	.79*	.62*	--	.45*	-.53*	*.31*	*.13*	.65*	*.09*
**PSS**	**- .02**	-**.32**	-**.18**	**-.08**	-**.17**	-**.02**	-**.12**	**.04**	**.15**	b	--	-.56*	.45*	*.05*	.62*	*.20*
**FPSC-A**	**- .04**	**.33**	**.11**	**.02**	-** .05**	**.09**	**.06**	-** .04**	-**.10**	-** .04**	-0.57*	–	-.71*	*-.22*	-.67*	*.00*
**FPSC-I**	**.13**	-**.30**	**-.04**	**.04**	**.10**	-** .07**	-**.01**	**.01**	**.14**	**.02**	.53*	b	-	*.17*	.52*	*.08*
**BCOPE-Ad**	**.06**	**-.37**	**.11**	**.14**	**.10**	**.10**	**.13**	**.11**	**.01**	-**.05**	-**.29**	.42*	-**0.42**	--	*.30*	*.12*
**BCOPE-M**	**.24**	**-.27**	**.16**	**.06**	**.02**	**.02**	**.08**	**.02**	**.14**	**-.09**	.54*	**-.32**	.45*	**-.05**	--	*.36*
**BCOPE-Al**	**-0.16**	**-.13**	**-.24**	**-.25**	**-.26**	**-.14**	**-.25**	**-.28**	**-.10**	**-.20**	**.28**	**-.18**	**.20**	**-.12**	**.04**	--

NB: *GAD* Generalised Anxiety Disorder symptoms, *SAD* Social Anxiety Disorder symptoms, *OCD* Obsessive Compulsive Disorder symptoms, *IUS* Intolerance of Uncertainty Scale, *ER* Emotion Regulation, *RBQ-ISS* Routined Behaviour Questionnaire- Insistence on Sameness Subscale, *PSS* Parent Stress Scale, *FPSC-A* Family Problem Solving Communication Index-Affirmatory, *FPSC-I* Family Problem Solving Communication Index-Incendiary, *BCOPE-Ad* Brief Coping Orientation to Problems Experienced-Adaptive, *BCOPE-M* Brief Coping Orientation to Problems Experienced-Maladaptive, *BCOPE-Al* Brief Coping Orientation to Problems Experienced-Alternative

^*^Significant difference between groups at *p* < .003 (Bonferroni adjustment)

Amongst both the DLD and typical samples, none of the correlations between age and total anxiety symptoms reached the Bonferroni-adjusted significance level (*ps* = 0.006–0.315), although there was a moderate positive correlation between age and total anxiety symptoms amongst the DLD sample (*r* = 0.34; older age indicated higher rate of anxiety).

For both samples, there were moderate to strong positive correlations between higher IU and higher anxiety across all anxiety and IU subscales (*r* = 0.48–0.76, *ps* < 0.003). This was particularly strong between social anxiety and the inhibitory subscale of the IUS for both the DLD sample (*r* (48) = 0.72, *p* < 0.003) and typical sample (*r* (42) = 0.73, *p* < 0.001). Insistence on sameness also had a moderate to strong significant correlation with all anxiety subtypes in both the DLD (*rs* = 0.49–0.61, *ps* < 0.003) and typical samples (*rs* = 0.54–0.79, *ps* < 0.003). For both samples, this correlation was strongest for total anxiety (DLD: r (42) = 0.65, *p* < 0.001; typical: r (41) = 0.79, *p* < 0.001) and OCD (DLD: r (42) = 0.61, *p* < 0.001; typical: r (41) = 0.77, *p* < 0.001). Higher preference for insistence on sameness had a stronger correlation with higher IU for the DLD sample (r (42) = 0.79, *p* < 0.003), than the typical sample (r (41) = 0.55, *p* < 0.001).

For the DLD sample only, higher emotion dysregulation had significant correlations with higher generalised anxiety (r (49) = 0.48, *p* < 0.003), social anxiety (r (49) = 0.46, *p* < 0.003) and, most strongly, separation anxiety scores (r (49) = 0.63, *p* < 0.001), but not OCD (r (49) = 0.33, *p* = 0.02). Similarly, ER, insistence on sameness and IU all had strong, positive and significant correlations with one another for the DLD sample (*r* = 0.62–0.79, *ps* < 0.003). In the typical sample, there was no significant correlation found between ER and either insistence on sameness (r (41) = 0.27, *p* = 0.09) or IU (r (42) = 0.34, *p* = 0.03), but there was a significant correlation between higher IU and increased preference for insistence on sameness (r (41) = 0.55, *p* < 0.001).

There was no significant correlation between the anxiety scales and either parenting stress or family communication styles within the DLD sample. Amongst the typical sample, however, both family communication styles and coping mechanisms had moderate to strong correlations with anxiety. An affirming communication style was significantly negatively correlated with separation anxiety (r (41) =  − 0.49, *p* < 0.003) as well as total anxiety (r (41) =  − 0.49, *p* < 0.003) and insistence on sameness (r (40) =  − 0.53, *p* < 0.001); more affirming communication correlated with lower anxiety and insistence on sameness. Conversely, maladaptive coping styles were significantly positively correlated with total anxiety (*rs* = 0.48–0.60, *ps* < 0.003); using more maladaptive coping strategies correlated with increased experiences of anxiety. Increased parent stress was also significantly positively correlated with increased insistence on sameness (r (41) = 0.45, *p* = 0.003), as well as increased incendiary communication (r (41) = 0.45, *p* = 0.003) and maladaptive coping styles (r (41) = 0.62, *p* < 0.003).

#### Mediation model

The mediation model sets out to explore the potential contributors towards the relationship between DLD diagnosis and anxiety. As IU, ISS and ER were found to have strong significant correlations with both variables, they were entered into the model as potential mediators.

Results from the parallel mediation analysis indicated that DLD diagnosis was indirectly related to anxiety through its relationship with both insistence on sameness and intolerance of uncertainty. As depicted in Fig. 2, children with DLD reported both higher ISS (*β* = 4.92, *SE* = 1.08, *LLCI* = 2.77, *ULCI* = 7.07, *p* < 0.001) and IU (*β* = 11.08, *SE* = 2.01, *LLCI* = 7.08, *ULCI* = 15.08, *p* < 0.001). In addition, both ISS and IU had significant positive relationships with anxiety (*β* = 1.85, *SE* = 0.49, *LLCI* = 0.16, *ULCI* = 1.27, *p* < 0.001; *β* = 0.54, *SE* = 0.23, *LLCI* = 0.37, *ULCI* = 0.98, *p* = 0.020, respectively). As such, when holding all other mediators constant, the indirect effect of DLD diagnosis on anxiety was significantly mediated through both ISS (*β* = 3.51, *SE* = 1.72, *LLCI* = 0.38, *ULCI* = 7.19, *z* = 2.91, *p* = 0.002) and IU (*β* = 7.47, *SE* = 2.44, *LLCI* = 3.13, *ULCI* = 12.79, *z* = 2.16, *p* = 0.015). Entering the mediators into the model increased its predictive power from 21.6 to 67.9%.

Also depicted in Fig. 2 is the relationship between DLD diagnosis and ER; children with DLD are more likely to experience emotional dysregulation (*β* = 4.19, *SE* = 0.87, *LLCI* = 2.46, *ULCI* = 5.93, *p* < 0.001). However, there was no significant relationship between ER and anxiety (*β* =  − 0.18, *SE* = 0.40, *p* = 0.65) and therefore no indirect effect of DLD diagnosis on anxiety through its relationship with ER (*β* = 0.99, *SE* = 1.52, *LLCI* =  − 1.96, *ULCI* = 4.08, *z* =  − 0.02, *p* = 0.490).

#### Qualitative insights

When presented with this model, parents were very enthused by it: “That is just Femi, that should be the tagline beneath his name… 100% you’ve nailed it… that makes perfect sense, it’s nice to see because it’s what I perhaps can’t articulate, but it’s so obvious.” (Josephine; Femi, 6 years old). Helen described how as follows:

For children like Joe, there is so little that they are able to control in life… without knowing what is expected of them or what’s happening, the fear of overload… I mean it must be terrifying, it must be like being partially sighted and expected to walk your way through a city you’ve never been to. (11 years old).

Helen went on to describe a pattern that Joe (11 years old) found himself in during the last year, “obsessing” over what happens in the afterlife, or what he refers to as “the spirit world”. She understood it as him trying to create some security, when the afterlife is perhaps the most unknown and uncontrollable phenomenon. She described how it almost drove her “nuts” as, for a year, “every evening he would have to rehearse a script with me around what will be in the spirit world… he had a whole written list of what he wanted…what it will look like”. Josephine and Liz (younger children; 6 and 7 years old, respectively) also described how they believe their children’s intolerances of uncertainty also contribute to their routined behaviours and preferences for sameness.

For Iris, model 1 made sense because she understood how Dimo’s DLD (10 years old) led to his feeling of uncertainty over day-to-day activities, an exhaustion of this uncertainty and thus resultant anxiety: “the fact that things go over his head, that is causing a certain level of uncertainty, because he can’t grasp it. And then of course it is only logical to slip into anxiety”. However, she also described how, for Dimo, this was experienced quite internally, and did not appear to drive his behaviours. For example, he never appears to seek out more information about upcoming situations in a proactive bid to feel more certain: “Not in a way of ‘if you don’t tell me what we’re doing tomorrow I am dreading tomorrow”.

## Discussion

This study aimed to estimate the prevalence of SEB difficulties amongst children with DLD, when compared to neurotypical children, and understand the mechanisms underlying the manifestation of anxiety. Significantly more parents of children with DLD reported problems across all SEB difficulties than those of typically developing children, providing further context to previous qualitative findings [16] and reinforcing wider quantitative literature (e.g. [109]).

Anxiety was found to be the most prevalent of all specified SEB difficulties for children with DLD, alongside insistence on sameness and emotion dysregulation; all of which shared significant correlations. Furthermore, the relationship between DLD diagnosis and anxiety was found to be fully mediated by IU and insistence on sameness. More specifically, the role of IU was most significant in fully mediating the relationship between DLD diagnosis and SAD. Emotion dysregulation only had a significant mediating effect on the relationship between DLD diagnosis and separation anxiety; insistence on sameness had no mediating effect on the relationship between DLD diagnosis and any of the anxiety subscales (SAD, GAD and separation anxiety). The models used in this investigation provide preliminary evidence for the direction of the relationships between the many different SEB difficulties experienced by children with DLD.

### The prevalence of socio-emotional and behavioural difficulties

The primary hypothesis of the study was that parents of children with DLD would report higher rates of all SEB difficulties than parents of typically developing children. Results supported this hypothesis and specified the difference to individual subdomains. This study highlights social anxiety and separation anxiety as specific targets for intervention; also characteristic of typically developing children of this age, though at lower rates [110]. Most other literature has focussed on “emotional difficulties” [10, 11, 111] or, more recently, “internalising symptoms” (e.g. [112]), negating the distinct contributing factors of specific symptoms. For example, a lack of “perceived controllability” has been associated with the development of separation anxiety [113], whereas negative self-appraisal significantly predicts social anxiety [114].

In focussing on these subdomains of “emotional difficulties”, this study suggests a relationship between age and onset of anxiety; that symptoms of social anxiety begin earlier than late adolescence [12], as previously suggested [115]. It also builds on previous work by suggesting that anxiety symptoms increase with age across childhood. For example, in typically developing populations, social communication difficulties have been found to significantly correlate with increases in social anxiety between ages 7 and 10 years old [42]. One suggestion for this pattern could be that anxiety increases as children become more aware of their differences (as in ASD; e.g. [116, 117]), as language requirements become more complex and their difficulties more obvious. Future studies should investigate possible links between self-awareness, social comparison, the manifestation of anxiety and the role that language plays in these relationships longitudinally.

The literature on preference for sameness and routine amongst children with DLD is in its infancy, yet it was highlighted as a key characteristic for most of the children in the DLD sample. The parents in the current study understood this as an attempt to “control” their environment, expanding on previous social findings [40]. Lloyd-Esenkaya and colleagues described a “structure” that children with DLD were observed to impose on their social interactions, illustrating a preference for sameness and routine within social activities [40]. It is understandable that the communication difficulties characteristic of DLD may well lead to feeling out of control of one’s environment; for example, the increased difficulties with using self-talk could limit their ability to process information about their surroundings or prepare for activities [118, 119]. Furthermore, speech and language therapists have described organisational and memory difficulties in many children with DLD [120]; the language difficulties mean these children may be less able to store additional information to help reassure them of upcoming uncertainty. Therefore, controlling their own environment through enforcing routine and sameness may be the most straightforward approach to managing their anxiety and frustration. More research is required to understand this preference amongst DLD populations more definitively.

Although omitted from the survey contents, the description of sensory sensitivity within our qualitative sample provides early context to other emerging work of children with DLD [121]. In children with other, more well-researched, NDDs [18], heightened sensory sensitivity has been suggested to both exacerbate social and separation anxiety [122], as well as provide some relief through calming strategies [123]. This is consistent with descriptions given by the parents in the current study, where sensory stimuli either triggered anxiety (e.g. too much quietness) or provided comfort from their anxieties (e.g. soft clothing). Furthermore, sensory sensitivity has been used to explain children’s tendencies towards sameness and routine in ASD populations [49, 122], as also demonstrated in this population. The fluctuating states of hypo and hyperarousal influence the child’s motivation towards sameness as an attempt at anxiety regulation [124]. More work needs to be done to investigate the manifestation of these behaviours amongst the DLD population.

In contrast to studies of other NDDs, sleep difficulties were relatively low, and emotional difficulties relatively high [125], emphasising the different needs of children with DLD, and requirement for novel intervention. The findings highlight the impact of internalising symptoms on families (e.g. “experiences anxiety”), in comparison to externalising symptoms (e.g. “has frequent tantrums”), and perhaps the need for more tailored intervention aimed at managing emotional difficulties (e.g. “Timid to Tiger”; [126]), rather than social and behavioural difficulties (e.g. “applied behavioural analysis”; [127]).

Two of the most polarising SEB difficulties for the DLD sample included statements pertaining to their social experience (i.e. “is often excluded from social situations”, “lacks awareness of others”). This echoes the heterogeneity in social skills found amongst children with DLD [7] and was further demonstrated by two mothers of boys at a similar age who described very different levels of social success: one had many friends (Dimo, 6 years old), whilst the other struggled to receive an invite to any playdates at all (Joe, 7 years old). The most prevalent of the social statements, “struggles to understand the intentions of others”, echoes the aforementioned emphasis given to the misinterpretation of social cues in qualitative work [16, 40]. There may be an opportunity for interventions to focus on nonlinguistic ways of interpreting interactions.

### The manifestation of anxiety in children with DLD

The second aim of this study looked to understand the mechanisms underlying the manifestation of anxiety. Families of typically developing children with symptoms of anxiety demonstrated more maladaptive coping mechanisms than families of children with DLD, as well as less affirming communication and higher parenting stress. All of these were significantly correlated with the child anxiety symptoms, supporting the wealth of literature linking these family characteristics with neurotypical SEB development (e.g. [128–131]). In comparison, families of children with DLD showed no such relationship. This is important, as it suggests that the manifestation of anxiety amongst children with DLD is less likely to be due to the parenting style or family factors. It is also remarkable, given the emotional strain that raising a child with NDDs can put on the family [132]. It is possible that the more developed communication abilities of the typical sample may mean they are more directly impacted by negative family communication styles (social learning theory; [133]). Instead, the emotional difficulties of children with DLD may be more likely to come more from cognitive differences, such as difficulties with ER due to their DLD [134]; this could increase a child’s likelihood of feeling overwhelmed and acting out behaviourally (e.g. [72]). As such, traditional parenting programmes (e.g. “Incredible Years”; [135]) may be less appropriate for DLD samples.

The relationship between DLD and anxiety subscales revealed specific correlation patterns. Intolerance of uncertainty significantly correlated with all anxiety subscales: GAD, SAD, OCD and separation anxiety. This relationship however was strongest for SAD. Given the interpersonal challenges that result from language difficulties (e.g. [136]), the children’s experience of uncertainty could likely be heightened in social contexts, rather than environmental contexts. In neurotypical samples, IU has been found to have a unique relationship with SAD but only regarding inhibitory anxiety (e.g. “when it’s time to act, uncertainty paralyses me” [53, 137]), rather than prospective anxiety (e.g. “I can’t stand being taken by surprise” [137, 138]). Evidence that SAD may manifest in the same way for children with DLD comes from the strong correlation found between SAD and the inhibitory subscale of IU.

Emotion dysregulation also had significant correlations with all anxiety subscales. This relationship was most pronounced for separation anxiety. It is possible that this could reflect the physical nature of separation anxiety when compared to the more cognitive emphasis of SAD in neurotypical children: children experience physical overwhelm when separating from their caregiver, rather than anticipating social rejection from peers (as in SAD; [139]). As in neurotypical samples, children experiencing separation anxiety could have further difficulties with low self-efficacy, or the reappraisal of negative emotional situations [140], processes that require inner speech and therefore language comprehension [33]. Furthermore, difficulties in expressing emotions through language may diminish the child’s receipt of necessary reassurance and encouragement from the caregiver, resulting in increased sensitivity towards separation. Children with DLD have expressed how their experience of safety and security is highly dependent on who they are with [46], further compounding the wish to stay with a trusted caregiver. Ensuring separation anxieties and emotional dysregulation are not carried into adulthood is important, as neurotypical adults who demonstrate these characteristics are suggested to have an increased risk of developing unhealthy interpersonal relationships [141].

The relationship between DLD and anxiety was found to be fully mediated by IU and insistence on sameness. Parents described how they felt their children experienced an overload of uncertainty on a daily basis due to their language difficulties, which resulted in an acute intolerance of any additional uncertainty (e.g. change in routine). This supports recent work by Jones and Westermann, demonstrating that children with DLD perceive a greater level of uncertainty in their environment when processing stimuli, due to their lack of coherent internal speech [142]. Worry, a behavioural characteristic of anxiety, has been identified as a cognitive strategy used to attempt to control the unknown [23, 143]. Furthermore, IU has been used to explain the avoidance behaviours of individuals with panic disorder [144]: individuals avoid unfamiliar situations in fear of experiencing the physical sensations of “panic” [144]; in other words, they insist on sameness. Research should investigate the relationship IU may have with similar behaviours exhibited by children with DLD, such as social withdrawal [7]. Similarly, the relationship between anxiety and insistence on sameness requires more investigation, as it could be bidirectional: used as a coping mechanism, as suggested amongst children with other NDDs (e.g. [145]).

This study also suggests that ER has a different association with anxiety than it does with depressive symptoms. The lack of mediation found between ER and anxiety is in contrast to prior literature suggesting emotion dysregulation mediates the relationship between DLD and depressive symptoms in slightly older children (8–15 years old; [14]). This supports what we know of neurotypical children; depressive symptoms that emerge later in childhood are due to their negative emotion processing styles [146]. Furthermore, it is consistent with previous literature that suggests emotion dysregulation has more of a role in the development of externalising symptoms, than internalising symptoms in children with DLD [13].

### Clinical implications

This study has clear implications for the treatment of SEB difficulties in children with DLD. Firstly, it highlights the need for anxiety management in this younger age group (6 to 12 years old), suggested through targeting an increase in children’s tolerance of uncertainty. Additionally, it suggests ER training could improve management of the children’s separation anxieties. Finally, the study has implications for the usefulness of different modes of intervention. Both the lack of familial influence, as well as the low prevalence of behavioural problems, suggests the potential irrelevance of “parent training” interventions (e.g. Cygnet: [147]; Triple P: [70]) and “behavioural training” interventions (e.g. Positive Plus Programme: [148]; Stop and Think: [149]). Instead, it may be more beneficial to base interventions on emotionally and cognitively focussed treatments (e.g. CUES: [150]) with clear awareness and consideration of the specific language needs of this population and adaptations thereof.

### Limitations

The context within which the survey was taken must be accounted for. In 2022, families were still recovering from the challenges of the Covid-19 global pandemic, a context recognised to increase the levels of anxiety amongst all children, neurotypical and those with NDDs (e.g. [125, 151]). Although these figures could be seen as inflated by the pandemic, the elevated rate of anxiety amongst those with DLD still highlights them as a vulnerable group and supports prior literature [5].

A further limitation is that confirmation of DLD diagnosis was parent-report only, without formal, independent assessment. Children were also not tested for their language abilities. The DLD population is recognised to be heterogenous [41], making it imperative that the individuals within any sample are confirmed to not just represent a specific subgroup. Nonetheless, it is this heterogeneity that renders uniform assessment challenging, with a general consensus lacking amongst professionals regarding the best method for assessing DLD [120]. Instead, the requirement of participants to have a “current” diagnosis of DLD builds on much of the DLD literature, which cites “children with a history of DLD” (e.g. [152]) or “children at risk of DLD” (e.g. [64]). Other studies have used previous definitions of DLD that excluded those with low IQ (e.g. [153]); children with lower IQ have been shown to have a higher risk of mental health difficulties at adolescence [154]. Relying on parent report of formal diagnosis is therefore more likely to be an accurate representation of the current DLD population.

The analysis conducted here was cross-sectional, and therefore, causal inferences cannot be drawn conclusively. For example, it is possible that experiencing anxiety could contribute towards preference for sameness as a way of coping with uncertainty [49]. Conversely, preference for sameness could either itself contribute to the manifestation of anxiety [155], or the relationship between the two could be bidirectional [156, 157]. Nonetheless, based on relevant theory (social adaptation model; [8]) and the developmental nature of DLD [158], the use of a mediation analysis suggests a directional relationship between DLD diagnosis and anxiety, IU, ER and ISS. As such, the analysis contributes to the theoretical basis from which the manifestation of anxiety can be understood through further longitudinal studies of children with DLD.

Finally, the “parent-informed SEB statements” were not validated but developed from statements provided by qualitative interviews of parents of children with DLD [16]. The measure was designed to capture the broad range of SEB difficulties experienced by children with DLD [159] whilst minimising survey length. Although measures validated in typically developing populations can indicate SEB symptoms amongst children with DLD, they are less likely to capture the full range of symptoms experienced [160]. Moreover, whilst the properties of SEB measures have been explored in children with other NDDs [161], there are no such explorations yet conducted for children with DLD. Future research should look to validate measures used amongst populations with DLD.

## Conclusions

This study adds to the literature on the SEB difficulties of children with DLD by suggesting some directional relationships between these difficulties and highlighting new characteristics that have been previously under-represented. Two of these characteristics are as follows: (1) a preference for routine and sameness and (2) an IU, both of which were found to be associated with one another as well as with symptoms of anxiety. Furthermore, IU and insistence on sameness were found to fully mediate the relationship between DLD diagnosis and anxiety, offering a preliminary insight into the manifestation of anxiety amongst these children. These findings alone provide a focus for the development of much needed intervention, preventing the escalation of these difficulties. It also suggests that there is a complex network of relationships between different SEB difficulties that exacerbate one another, which future work should look to disentangle.

## Data Availability

Anonymised quantitative data analysed during the current study is available from the corresponding author upon reasonable request.

## Abbreviations

ASD: Autism Spectrum Disorder
Brief-COPE: Brief-Coping Orientation to Problems Experienced
DLD: Developmental Language Disorder
E-DLD: Engage with developmental language disorder
ER: Emotion regulation
FPSC: Family Problem-Solving Communication Index
GBP: Great British Pound
ISS: Insistence on sameness subscale
IU: Intolerance of uncertainty
IUS: Intolerance of uncertainty scale
NDD: Neurodevelopmental disorders
PSS: Parenting Stress Scale
RBQ: Repetitive Behaviour Questionnaire
SCAS-P: Spence Children’s Anxiety Scale
SEB: Socio-emotional and behavioural
UK: United Kingdom
