# Correction: Understanding the prevalence and manifestation of anxiety and other socio‑emotional and behavioural difficulties in children with Developmental Language Disorder

**DOI:** 10.1186/s11689-023-09488-8

**Published:** 2023-06-30

**Authors:** Annabel Burnley, Michelle St Clair, Rachael Bedford, Yvonne Wren, Charlotte Dack

**Affiliations:** 1 Department of Psychology, University of Bath, Bath, Somerset County UK; 2 Bristol Dental School, University of Bristol, Bristol, Bristol County UK

**Correction: J Neurodev Disord 15, 17 (2023)**

**https://doi.org/10.1186/s11689-023-09486-w**

Following publication of the original article [1], the authors’ identified an error on Fig. 1. There are asterisks (*) missing from the chart that would denote significant differences. The correct Fig. 1 is and the original article [1] has been corrected.

**Fig. 1:**
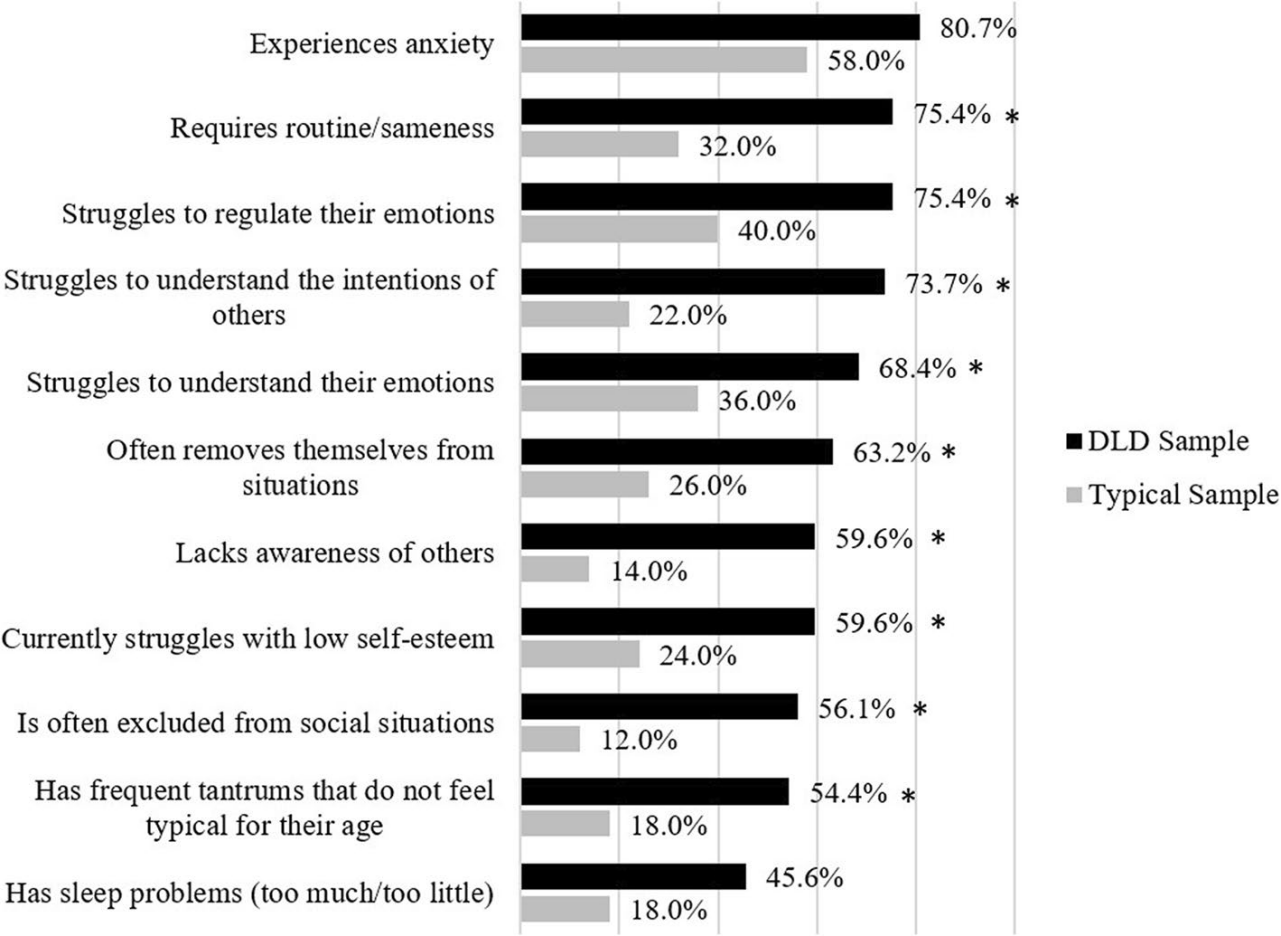
Difference between groups on parent’s self-report of their child’s psychosocial difficulties *Significant difference between groups at *p* < .005 (Bonferroni adjustment)
